# The Long-Term Outcome of Laparoscopic Resection for Perihilar Cholangiocarcinoma Compared with the Open Approach: A Real-World Multicentric Analysis

**DOI:** 10.1245/s10434-022-12647-1

**Published:** 2022-10-22

**Authors:** Tingting Qin, Min Wang, Hang Zhang, Jingdong Li, Xiaxing Deng, Yuhua Zhang, Wenxing Zhao, Ying Fan, Dewei Li, Xuemin Chen, Yechen Feng, Siwei Zhu, Zhongqiang Xing, Guangsheng Yu, Jian Xu, Junjie Xie, Changwei Dou, Hongqin Ma, Gangshan Liu, Yue Shao, Weibo Chen, Simiao Xu, Jun Liu, Jianhua Liu, Xinmin Yin, Renyi Qin, Minimally Invasive Pancreas Treatment Group in the Pancreatic Disease Branch of China’s International Exchange and Promotion Association for Medicine and Healthcare

**Affiliations:** 1 Department of Biliary–Pancreatic Surgery, Affiliated Tongji Hospital, Tongji Medical College, Huazhong University of Science and Technology, Wuhan, Hubei China; 2 Department of Hepatobiliary Surgery, Affiliated Hospital of North Sichuan Medical College, Hepatobiliary, Pancreatic and Intestinal Diseases Research Institute of North Sichuan Medical College, Nanchong, China; 3 Department of Ruijin Hospital, Shanghai Jiao Tong University School of Medicine, Shanghai, China; 4 Department of Hepatobiliary, Pancreatic and Minimal Invasive Surgery, Zhejiang Provincial People’s Hospital, People’s Hospital of Hangzhou Medical College, Hangzhou, 310003 China; 5 Department of General Surgery, The Affiliated Hospital of Xuzhou Medical University Xuzhou, Jiangsu, China; 6 Department of the Second General Surgery, Sheng Jing Hospital of China Medical University, Liaoning, China; 7 Department of Hepatobiliary Surgery, The First Affiliated Hospital of Chongqing Medical University, Chongqing, China; 8 Department of Hepatopancreatobiliary Surgery, The Third Affiliated Hospital of Soochow University, Suzhou, China; 9 Department of Hepatobiliary Surgery, Hunan Provincial People’s Hospital, The First Affiliated Hospital of Hunan Normal University, Changsha, Hunan China; 10 Department of Hepato-Pancreato-Biliary Surgery, The Second Hospital of Hebei Medical University, Shijiazhuang, Hebei China; 11 Shandong Provincial Institute of Dermatology and Venereology, Shandong Academy of Medical Sciences, Shandong Provincial Hospital Affiliated to Shandong University, Jinan, Shangdong China; 12 Department of Endocrinology, Affiliated Tongji Hospital, Tongji Medical College, Huazhong University of Science and Technology, Wuhan, China

## Abstract

**Objective:**

The aim of this study was to compare the short- and long-term outcomes of laparoscopic surgery (LS) and open surgery (OP) for perihilar cholangiocarcinoma (PHC) using a large real-world dataset in China.

**Methods:**

Data of patients with PHC who underwent LS and OP from January 2013 to October 2018, across 10 centers in China, were extracted from medical records. A comparative analysis was performed before and after propensity score matching (PSM) in the LS and OP groups and within the study subgroups. The Cox proportional hazards mixed-effects model was applied to estimate the risk factors for mortality, with center and year of operation as random effects.

**Results:**

A total of 467 patients with PHC were included, of whom 161 underwent LS and 306 underwent OP. Postoperative morbidity, such as hemorrhage, biliary fistula, abdominal abscess, and hepatic insufficiency, was similar between the LS and OP groups. The median overall survival (OS) was longer in the LS group than in the OP group (NA vs. 22 months; hazard ratio [HR] 1.19, 95% confidence interval [CI] 1.02–1.39, *p* = 0.024). Among the matched datasets, OS was comparable between the LS and OP groups (NA vs. 35 months; HR 0.99, 95% CI 0.77–1.26, *p* = 0.915). The mixed-effect model identified that the surgical method was not associated with long-term outcomes and that LS and OP provided similar oncological outcomes.

**Conclusions:**

Considering the comparable long-term prognosis and short-term outcomes of LS and OP, LS could be a technically feasible surgical method for PHC patients with all Bismuth–Corlett types of PHC.

**Supplementary Information:**

The online version contains supplementary material available at 10.1245/s10434-022-12647-1.

Perihilar cholangiocarcinoma (PHC) is one of the most dismal malignancies involving the confluence of the hepatic ducts, and contributes to >50% of malignant tumors of the biliary tract.^1,2^ Owing to its insensitivity to radiotherapy and chemotherapy, surgical resection has become a potentially curative therapeutic option for PHC.^3^ However, due to its aggressiveness, late presentation, and refractory nature, most patients are admitted following a late clinical diagnosis, with macrovascular invasion, lymph node or liver parenchyma involvement, resulting in only 10–15% of patients being eligible for resection with curative intent.^4,5^ Even after resection, the 5-year survival rate remains disappointing at around 10–35%.^6–8^

The surgical difficulty for PHC is known as the Mount Everest of Abdominal Surgery.^9^ Conventional radical resection of PHC includes complete resection of the extrahepatic bile duct, extended hemihepatectomy with complete caudate lobectomy, lymph node dissection in the hepatoduodenal ligament, and choledochojejunostomy.^10,11^ With improvements in laparoscopic techniques and the gradual establishment of laparoscopic surgical procedures, advancements in laparoscopic resection have revolutionized the process for most abdominal surgeries, including resection for colorectal cancer,^12–14^ pancreatoduodenectomy,^15,16^ all types of hepatobiliary resections,^17–20^ and cholangiocarcinoma surgery.^21^ Laparoscopic surgery (LS) for PHC has been shown to be safe and feasible, with comparable short-term outcomes as open surgery (OP).^22,23^ However, most LSs for PHC are limited to carefully selected patients and are technically achievable for experienced surgeons. High-volume and comparative studies are lacking, and the evidence is undoubtedly biased.^24^ Moreover, the long-term outcome of LS for PHC is lacking owing to the limited laparoscopic experience and the absence of long-term follow-up as this is a newly applied technique.

It is imperative to undertake large-scale multicenter analyses to investigate the technical feasibility and safety of LS for PHC. Therefore, we performed a multicenter real-world study to compare the long-term survival of PHC patients who underwent LS or OP, to summarize the updated applications, advancements, and limitations of LS for treating PHC and help with decision making during treatment.

## Materials and Methods

### Patient Selection

A retrospective review of real-world institutional databases from 10 hospitals in China identified 467 patients with PHC who underwent curative surgery (including R0 and R1) from January 2013 to October 2018. Patients with pathologically confirmed PHC and no evidence of distant metastasis on preoperative examination were included. Those who underwent combined hepato-pancreaticoduodenal resection, extrahepatic bile duct resection only, were lost to follow-up, or had missing data on the main outcomes, were excluded. All included cases met the resectability criteria laid down by the National Comprehensive Cancer Network guidelines for preoperative assessments.^25^ All cases were systematically discussed during multidisciplinary hepatobiliary meetings, involving experienced surgeons, radiologists, endoscopists, oncologists, radiation specialists, and pathologists, to define the indications and characteristics of the surgical procedure and to share the steps of perioperative optimization. This study was approved by the Ethics Committee of Tongji Hospital (approval number TJ-IRB20220531). This work has been reported in line with the STROCSS (Strengthening the Reporting of Cohort Studies in Surgery) criteria.^26^ All participating centers were high-volume hepatic surgical centers, and the surgical teams were experienced in both LS and OP.

### Surgical Technique and Follow-Up

Preoperatively, all patients underwent three-dimensional visualization to clearly show the intrahepatic pipeline, size and location of tumors, and the relationship between the tumor and intrahepatic pipeline. LS was defined as a total laparoscopic surgery. Surgical procedures included hepatectomy with *en bloc* resection of the caudate lobe and extrahepatic bile duct as well as regional lymph node dissection. The procedure for PHC included (1) local excision of hilar bile ducts only; (2) left hemihepatectomy; (3) right hemihepatectomy; (4) extended left hemihepatectomy; (5) extended right hemihepatectomy; and (6) segmentectomy (≤3 Couinaud segments). Frozen section examination of the proximal and distal bile duct resection margins was routinely performed intraoperatively. The surgical techniques, steps, and principles of surgery are similar between the laparoscopic and open approaches.

### Data Collection and Definitions

Baseline characteristics included patient age, sex, body mass index, American Society of Anesthesiologists score,^27^ year of operation, tumor differentiation, M stage, T stage, N stage, TNM stage, and history of adjuvant treatment. The TNM staging was based on the American Joint Committee on Cancer (AJCC) staging system (8th edition).^28^ The primary endpoint was long-term overall survival (OS) after initial radical surgery, which was defined as the duration from the first postoperative day to either the date of death or last follow-up. Secondary endpoints were perioperative outcomes, including postoperative complications, reoperation, mortality within 30 and 90 days, readmission within 90 days, and postoperative length of stay (LOS). Postoperative complications were reviewed within 90 days after surgery and were graded according to the Clavien–Dindo (CD) classification system.^29^ Postoperative biliary leakage,^30^ hemorrhage,^31^ and liver failure^32^ were defined and classified according to the criteria established by the International Study Group of Liver Surgery (ISGLS). Wound infection was defined as purulent drainage from the incision and/or positive culture findings of the fluid or tissue aseptically obtained from the incision. Operative details, including operation duration, intraoperative blood loss (IBL), blood transfusion, vascular resection, number of resected lymph nodes, and R0 resection, were also analyzed. R0 resection was defined as tumor-free margins in all reported surgical margins (biliary and circumferential). The definitions of all these parameters were unified by all participating teams at the beginning of this study. All patients were recommended to return for follow-up at the outpatient department 1 month after discharge, every 3–6 months for the first 2 years, and annually thereafter. Survival data were collected by searching the electronic outpatient system or by telephone interviews. Final follow-up was conducted in January 2020.

### Statistical Analyses

To minimize the potential bias from confounding factors between the OP and LS groups in real-world data, propensity matching was performed to create a pseudo-randomized population. LS and OP were matched 1:1 using the nearest-neighbor matching method without replacement. A caliper radius equal to a standard deviation (SD) of 0.1 was set to prevent poor matching.

Continuous variables were expressed as medians and interquartile ranges (IQRs) or mean (SD), while categorical variables were expressed as numbers (*n*) and percentages (%). Independent-samples *t*-tests were performed to compare continuous variables that followed normal distributions; otherwise, the Mann–Whitney U test was used. Chi-square or Fisher’s exact tests were used to compare categorical variables. Survival analyses were conducted using the Kaplan–Meier method with log-rank tests. Multivariate Cox regression analyses were used to estimate the risk factors for long-term all-cause mortality. To further consider the measures of hidden confounders, the period of operation (2013–2018) and the centers (e.g., improved perioperative care in recent years and different surgical management in different hospitals) were included as random effects in the mixed-effects Cox regression model. The results are presented as hazard ratios (HRs) with corresponding 95% confidence intervals (CIs). The proportional hazards assumption of the Cox proportional regression model was assessed by eyeballing the Kaplan–Meier plot and the log-minus-log plot.^33^ All statistical procedures were conducted using SAS software version 9.40 (SAS Institute, Inc., Cary, NC, USA). Two-sided hypothesis testing with a predetermined level of *p* < 0.05 was considered statistically significant.

## Results

### Patient Clinicopathological Characteristics and Pathologic Features

**Fig. 1:**
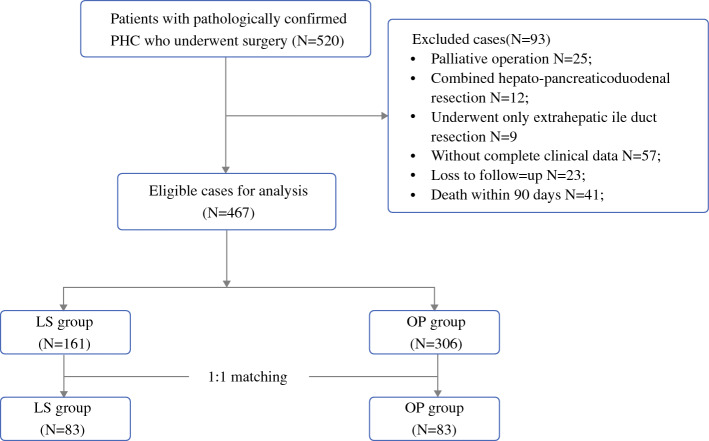
Study flow chart. *PHC* perihilar cholangiocarcinoma, *LS* laparoscopic surgery, *OP* open surgery

Among the 467 PHC patients, 161 underwent LS and 306 underwent OP (Fig. 1). The percentage of LS in these hospitals increased from 36.36% (12 of 33) in 2013 to 38.24% (26 of 48) in 2018. The median follow-up period was 24 months (IQR 13–75) for the entire cohort, 21 months (IQR 13–32) for the LS cohort, and 28 months (IQR 14–42) for the OP cohort. In the original cohort, preoperative liver function indices, such as total bilirubin (Tbil), aspartate aminotransferase (AST), and alanine aminotransferase (ALT), were significantly lower in the LS group. A higher TNM stage (TVa and TVb) was observed in the OP group (*p* < 0.001). There was no statistical difference in the Bismuth–Corlett types between LS and OP, with the following details: I in 16 (3.4%), IIA in 108 (23.1%), IIB in 102 (21.8%), IIIA in 44 (9.4%), IIIB in 61 (13.1%), and IV in 134 (26.7%) patients. After propensity score matching (PSM), 83 patients in the OP group were well matched with 83 patients in the LS group and comprised the matched cohort. The baseline data are shown in Table 1.

**Table 1:** Baseline, preoperative, and oncological characteristics

Characteristic	Before PSM			After PSM		
	LS [*n* = 161]	OP [*n* = 306]	*p*-value	LS [*n* = 83]	OP [*n* = 83]	*p*-value
*Patient*						
Age, years [mean (SD)]	62.99 (9.32)	62.20 (9.46)	0.390	62.06 (9.44)	62.67 (10.30)	0.689
Female [*n* (%)]	77 (47.83)	116 (37.91)	0.039	43 (51.81)	39 (46.99)	0.535
BMI, kg/m^2^ [mean (SD)]	22.93 (3.18)	22.66 (2.70)	0.329	22.65 (3.11)	22.92 (2.76)	0.552
ASA score [*n* (%)]						
I	37 (22.98)	70 (22.88)	0.986	26 (31.33)	27 (32.53)	0.861
II	100 (62.11)	192 (62.75)		47 (56.63)	44 (53.01)	
III	24 (14.91)	44 (14.38)		10 (12.05)	12 (14.46)	
Tbil, μmol/L [median (IQR)]	48.40 (43.70–56.40)	124.85 (80.20–218.70)	< 0.0001	51.60 (42.90–93.00)	72.70 (35.70–112.50)	0.780
AST, μmol/L [median (IQR)]	45.10 (34.30–98.50)	99.00 (57.00–171.50)	< 0.0001	62.40 (36.00–118.20)	80.30 (45.30–122.00)	0.869
ALT, μmol/L [median (IQR)]	63.30 (32.00–144.20)	126.90 (62.00–203.00)	< 0.0001	82.50 (32.20–155.70)	116.00 (61.00–163.20)	0.621
Albumin, μmol/L [median (IQR)]	35.70 (33.70–38.50)	36.05 (33.40–39.10)	0.982	35.30 (33.40–38.00)	35.90 (33.00–39.00)	0.671
CA199, U/mL [median (IQR)]	191.40 (72.70–612.00)	238.00 (98.50–418.90)	0.803	199.30 (62.60–399.30)	185.40 (69.80–385.60)	0.463
Year of operation [*n* (%)]						
2013–2014	25 (15.53)	69 (22.55)	0.051	12 (14.46)	19 (22.89)	0.039
2015–2016	53 (32.92)	113 (36.93)		28 (33.73)	37 (44.58)	
2017–2018	83 (51.55)	124 (40.52)		43 (51.81)	27 (32.53)	
*Tumor oncological characteristics*						
Maximun tumor size, cm [median (IQR)]	2.80 (2.00–3.50)	2.80 (2.00–3.50)	0.708	2.50 (2.00–3.50)	3.00 (2.00–3.50)	0.687
Bismuth–Corlett type [*n* (%)]						
I	4 (2.50)	12 (3.93)	0.818	2 (2.41)	3 (3.61)	0.356
IIa	41 (25.63)	67 (21.97)		19 (22.89)	19 (22.89)	
IIb	36 (22.50)	66 (21.64)		22 (26.51)	12 (14.46)	
IIIa	17 (10.63)	27 (8.85)		5 (6.02)	11 (13.25)	
IIIb	19 (11.88)	42 (13.77)		11 (13.25)	12 (14.46)	
IV	43 (26.88)	91 (29.84)		24 (28.92)	26 (31.33)	
AJCC TNM stage [*n* (%)]			0.001			0.992
I (T1N0M0)	19 (12.10)	59 (19.73)		14 (16.87)	15 (18.07)	
II (T2a/2bN0M0)	73 (46.50)	122 (40.80)		38 (45.78)	39 (46.99)	
IIIA (T3N0M0)	25 (15.92)	21 (7.02)		9 (10.84)	7 (8.43)	
IIIB (T4N0M0)	14 (8.92)	15 (5.02)		5 (6.02)	4 (4.82)	
IVA (T, N2M0)	20 (12.74)	56 (18.73)		12 (14.46)	12 (14.46)	
IVB (T,N,M1)	6 (3.82)	26 (8.70)		5 (6.02)	6 (7.23)	

*PSM* propensity score matching, *LS* laparoscopic surgery, *OP* open surgery, *SD* standard deviation, *BMI* body mass index, *ASA* American Society of Anesthesiologists, *Tbil* total bilirubin, *AST* aspartate aminotransferase, *ALT* alanine aminotransferase, *IQR* interquartile range, *AJCC* American Joint Committee on Cancer

### Operative Characteristics and Related Short-Term Outcomes

The surgical characteristics of the patients and the related short-term outcomes in the LS and OP groups are shown in Table 2. In the original cohort, patients who underwent LS had comparable operative time (median, 350 vs. 345 min, *p* = 0.054), blood loss (median, 250 vs. 300 mL, *p* = 0.859), and R0 resection (91.3% vs. 91.8%, *p* = 0.845) with OP. However, more enlarged hepatectomy, less vascular resection, and less biliary plasty were observed in the LS group. Furthermore, a higher percentage of people received postoperative chemotherapy in the LS group compared with the OP group (44.72% vs. 15.36%, *p* < 0.0001). In the matched cohort, except for less vascular resection observed in the LS group, the other operative characteristics were comparable between the two groups.

**Table 2:** Intraoperative characteristics and postoperative outcomes

Variable	Before PSM			After PSM		
	LS [*n* = 161]	OP [*n* = 306]	*p*-value	LS [*n* = 83]	OP [*n* = 83]	*p*-value
Operative time, min [median (IQR)]	350.0 (280.0–420.0)	345.0 (284.0–395.0)	0.051	360.0 (300.0–420.0)	356.0 (300.0–400.0)	0.914
Transfusion during surgery [*n* (%)]	45 (28.0)	104 (34.0)	0.184	21 (25.3)	26 (31.3)	0.389
Transfusion volume, mL [median (IQR)]	0.0 (0.0–400.0)	0.0 (0.0–600.0)	0.838	0.0 (0.0–200.0)	0.0 (0.0–400.0)	0.957
EBL, mL [median (IQR)]	250.0 (100.0–500.0)	300.0 (200.0–500.0)	0.859	300.0 (100.0–500.0)	300.0 (100.0–500.0)	0.775
No. of harvested lymph nodes [median (IQR)]	8.0 (5.0–8.0)	7.0 (5.00–10.0)	0.122	8.0 (5.0–10.0)	8.0 (5.0–10.0)	0.570
R0 resection [*n* (%)]	147 (91.3)	281 (91.8)	0.845	78 (94.0)	73 (87.9)	0.176
Hepatectomy [*n* (%)]						
Bile duct only	60 (37.3)	127 (41.5)	0.049^a^	29 (34.9)	30 (36.1)	0.857^a^
Left hemihepatectomy	74 (46.0)	121 (39.5)		41 (49.4)	34 (41.0)	
Right hemihepatectomy	14 (8.7)	48 (15.7)		8 (9.6)	12 (14.5)	
Left segmentectomy	2 (1.2)	3 (1.0)		1 (1.2)	1 (1.2)	
Right segmentectomy	3 (1.9)	2 (0.7)		1 (1.2)	2 (2.4)	
Bile duct and part of hepatectomy	8 (5.0)	5 (1.6)		3 (3.6)	4 (4.8)	
Conversion to laparotomy [*n* (%)]	14 (8.7)	0 (0.0)		9 (10.8)	0 (0.0)	
Vascular resection [*n* (%)]			< 0.0001^a^			0.018^a^
None	148 (91.9)	230 (75.2)		79 (95.2)	70 (84.3)	
Hepatic artery	9 (5.6)	18 (5.9)		3 (3.6)	2 (2.4)	
Portal vein	3 (1.9)	9 (2.9)		1 (1.2)	2 (2.4)	
Hepatic artery and portal vein	1 (0.6)	49 (16.0)		0 (0.0)	9 (10.8)	
Digestive reconstruction [*n* (%)]			< 0.0001			0.987
Choledochojejunostomy	77 (47.8)	90 (29.4)		31 (37.4)	32 (38.6)	
Hepaticojejunostomy	70 (43.5)	208 (68.0)		48 (57.8)	47 (56.6)	
Biliary plasty [*n* (%)]	56 (34.8)	173 (56.5)	< 0.0001	44 (53.0)	47 (56.6)	0.640
Put a stent in [*n* (%)]	10 (6.2)	24 (7.8)	0.519	6 (7.2)	9 (10.8)	0.417
Chemotherapy [*n* (%)]	72 (44.7)	47 (15.4)	< 0.0001	23 (27.7)	21 (25.3)	0.725
Overall postoperative complications [*n* (%)]						
Biliary fistula [*n* (%)]	14 (8.70)	24 (7.8)	0.749	8 (9.64)	7 (8.43)	0.787
Hemorrhage [*n* (%)]	14 (8.70)	14 (4.6)	0.075	4 (4.82)	1 (1.20)	0.173^a^
CD stage ≥III [*n* (%)]	21 (13.0)	76 (24.8)	0.003	10 (12.1)	16 (19.3)	0.200
Death, 30 days [*n* (%)]	8 (5.0)	21 (6.9)	0.420	6 (7.2)	6 (7.2)	> 0.999
Death, 90 days [*n* (%)]	12 (7.5)	29 (9.5)	0.463	7 (8.4)	6 (7.2)	0.773
Postoperative hospital stay, days [median (IQR)]	13.0 (10.0–19.0)	15.0 (12.0–22.0)	0.0006	14.0 (10.0–19.0)	15.0 (12.0–22.0)	0.022

*PSM* propensity score matching, *LS* laparoscopic surgery, *OP* open surgery, *IQR* interquartile range, *EBL* estimated blood loss, *CD* Clavien–Dindo

^a^Fisher’s exact test

The median postoperative LOS was shorter (13 vs*.* 15 days, *p* = 0.0006) and the overall postoperative morbidity rate was significantly lower in the LS group than in the OP group (31.1% vs. 41.5%, *p* = 0.027), with severe complications (CD ≥III) accounting for 13.0% in the LS group compared with 24.8% in the OP group (*p* = 0.003). Major postoperative complications such as biliary fistula and hemorrhage were comparable between the two groups. After matching, the complication and reoperation rates were similar between the two groups. Overall, the LS group was associated with significant LOS reduction (median, 14 vs. 15 days, *p* = 0.022) and a shorter postoperative drainage tube keep (PDTK) time (median, 6 vs. 8 days, *p* = 0.035) than the OP group.

### Long-Term Outcomes and Overall Survival

**Fig. 2:**
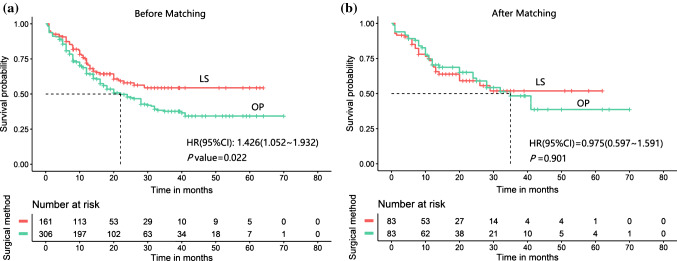
Kaplan–Meier curves for overall survival of PHC patients undergoing LS versus OP. **a** Before propensity score matching; **b** propensity score matching. *PHC* perihilar cholangiocarcinoma, *LS* laparoscopic surgery, *OP* open surgery, *HR* hazard ratio, *CI* confidence interval

**Fig. 3:**
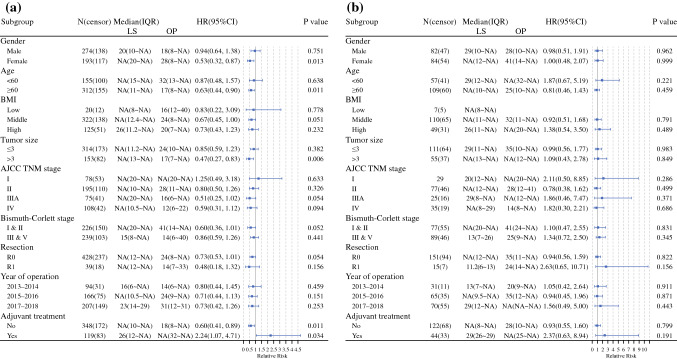
Forest plot of risk evaluation of LS compared with OP for PHC patients in different subgroups. **a** Before matching; **b** after matching. *PHC* perihilar cholangiocarcinoma, *LS* laparoscopic surgery, *OP* open surgery, *IQR* interquartile range, *HR* hazard ratio, *CI* confidence interval, *BMI* body mass index, *AJCC* American Joint Committee on Cancer

By October 2018, 212 patients (45.4%) had died. The 90-day mortality rate was 7.45% (12 of 161) in the LS cohort and 9.48% (29 of 306) in the OP cohort. These 41 patients were excluded from the long-term mortality analysis. The 1-year (72.2% vs. 64.6%) and 2-year (57.9% vs. 48.0%) OS rates in the LS group were higher than those in the OP group (*p* < 0.022) before matching. The median OS was NA months in the LS group and 22 months in the OP group (HR 1.19, 95% CI 1.02–1.39, *p* = 0.024) (Fig. 2a). After matching, the year-specific survival rates were similar between the two groups (1-year [71.3% vs. 70.2%] and 2-year [52.1% vs. 48.4%], with similar mortality observed in the LS and OP groups [log-rank test, *p* = 0.912]). The median OS was NA and 35 months in the LS and OP groups, respectively (HR 0.99, 95% CI 0.77–1.26, *p* = 0.915) [Fig. 2b]. In addition, the OS of the two surgical groups was also stratified by subgroups, and the results showed that females and patients older than 60 years of age can benefit from LS surgery (Fig. 3a). When patients were well-matched, the surgical outcomes did not differ between the two surgical groups (Fig. 3b). The year-specific survival rates for most subgroup characteristics are shown in electronic supplementary Table S1.

**Fig. 4:**
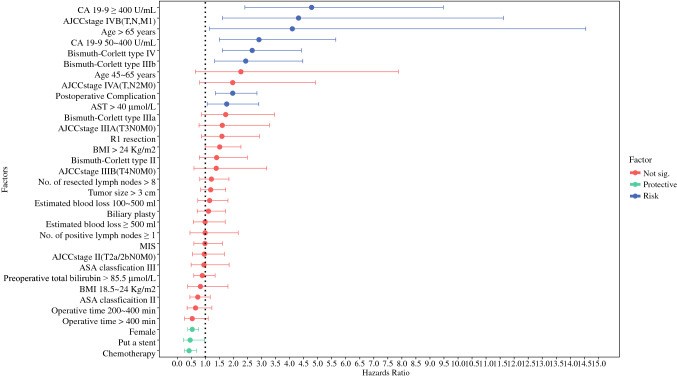
Fixed-effects estimate for OS predictors. The strongest patient-factor predictors are higher CA19-9 level, higher AJCC stage, older age, higher Bismuth–Corlett type, had postoperative complication, and higher level of AST. *OS* overall survival, *AJCC* American Joint Committee on Cancer, *AST* aspartate aminotransferase, *BMI* body mass index, *ASA* American Society of Anesthesiologists

Table 3 shows the results of univariate and multivariate Cox proportional hazards mixed-effects models for OS, with center and year of operation as the random effects. The proportional hazard assumption was not violated (*p* = 0.224). The estimated coefficients for each predictor are shown in Fig. 4. Higher AST levels, Bismuth–Corlett type, CA19-9 levels, age >65 years, and higher AJCC stage were key risk predictors of long-term survival (HR 1.7 to +4.8). Being female, receiving a stent, and receiving chemotherapy were key protective predictors (HR 0.4 to +0.6). Compared with these fixed effects, the between-center variation (HR 0.5 to +2.1) and between-year variation (HR 0.8 to +1.1) were much smaller than the fixed-effects predictors (electronic supplementary Fig. S1).

**Table 3:** Hazard ratios for crude and adjusted association between risk factors and long-term mortality (excluding 30-day mortality) under the Cox-adjusted model and propensity score-matched model

Characteristic	Before matching				After matching	
	Crude		Adjusted		Adjusted	
	HR (95% CI)	*p*-value^a^	HR (95% CI)	*p*-value^b^	HR (95% CI)	*p*-value^c^
Age, years						
<45	Reference		Reference		Reference	
45–65	2.22 (0.65–7.59)	0.204	2.27 (0.65–7.88)	0.200	3.2 (0.28–36.5)	0.350
>65	4.03 (1.16–14.02)	0.028	4.1 (1.15–14.55)	0.029	7.6 (0.63–91.06)	0.110
Female	0.56 (0.4–0.79)	0.001	0.53 (0.37–0.76)	0.001	0.63 (0.29–1.38)	0.240
ASA						
I	Reference		Reference		Reference	
II	0.83 (0.53–1.3)	0.408	0.73 (0.45–1.17)	0.180	0.98 (0.33–2.91)	0.970
III	1.15 (0.62–2.15)	0.655	0.95 (0.49–1.85)	0.890	0.92 (0.19–4.49)	0.920
BMI, kg/m^2^						
<18.5	Reference		Reference		Reference	
18.5–24	0.79 (0.36–1.71)	0.544	0.82 (0.37–1.81)	0.620	0.42 (0.04–4.67)	0.480
>24	1.43 (0.97–2.12)	0.071	1.51 (0.99–2.27)	0.051	1.15 (0.47–2.78)	0.760
Tbil >85.5 μmol/L	0.96 (0.65–1.44)	0.860	0.89 (0.59–1.35)	0.590	0.61 (0.24–1.56)	0.300
AST >40 μmol/L	1.53 (0.93–2.5)	0.094	1.76 (1.06–2.9)	0.028	3.05 (1.01–9.17)	0.047
Tumor oncology						
Tumor size >3 cm	1.16 (0.81–1.67)	0.411	1.19 (0.83–1.72)	0.350	1.23 (0.52–2.89)	0.640
CA199, U/mL						
≤50	Reference		Reference		Reference	
50–400	2.62 (1.37–5.03)	0.004	2.91 (1.5–5.64)	0.002	7.94 (2.04–30.92)	0.003
≥400	4.75 (2.41–9.36)	0.000	4.78 (2.41–9.48)	0.000	21.93 (4.73–101.67)	0.000
No. of resected lymph nodes >8	1.2 (0.83–1.75)	0.334	1.21 (0.8–1.84)	0.370	0.82 (0.32–2.08)	0.680
No. of positive lymph nodes ≥1	0.84 (0.4–1.77)	0.648	0.99 (0.45–2.17)	0.990	2.87 (0.38–21.62)	0.310
Bismuth–Corlett type						
I	Reference		Reference		Reference	
II	1.4 (0.8–2.44)	0.238	1.4 (0.79–2.5)	0.250	2.63 (0.68–10.07)	0.160
IIIa	1.45 (0.75–2.8)	0.269	1.73 (0.87–3.46)	0.120	2.84 (0.65–12.41)	0.170
IIIb	2.07 (1.16–3.7)	0.014	2.44 (1.33–4.47)	0.004	3.32 (0.92–12.00)	0.067
IV	2.26 (1.38–3.69)	0.001	2.67 (1.61–4.42)	0.000	5.19 (1.76–15.31)	0.003
AJCC TNM stage						
I (T1N0M0)	Reference		Reference		Reference	
II (T2a/2bN0M0)	1.04 (0.6–1.81)	0.892	0.96 (0.54–1.68)	0.880	0.5 (0.15–1.63)	0.250
IIIA (T3N0M0)	1.65 (0.81–3.35)	0.169	1.6 (0.78–3.28)	0.200	1.33 (0.28–6.35)	0.720
IIIB (T4N0M0)	1.65 (0.73–3.72)	0.231	1.38 (0.6–3.19)	0.450	0.57 (0.06–5.28)	0.620
IVA (T, N2M0)	2.29 (0.94–5.56)	0.068	1.98 (0.8–4.92)	0.140	0.76 (0.08–7.62)	0.820
IVB (T,N,M1)	4.15 (1.57–10.98)	0.004	4.32 (1.6–11.62)	0.004	1.6 (0.13–20.31)	0.720
*Surgical approach*						
LS	1.08 (0.68–1.71)	0.748	0.98 (0.59–1.62)	0.930	1.37 (0.64–2.94)	0.420
Operative time, min						
≤200	Reference		Reference		Reference	
200–400	0.7 (0.38–1.3)	0.263	0.65 (0.35–1.23)	0.180	0.29 (0.07–1.21)	0.089
>400	0.55 (0.27–1.12)	0.101	0.53 (0.26–1.1)	0.087	0.18 (0.03–1.04)	0.055
Estimated blood loss, mL						
≤100	Reference		Reference		Reference	
100–500	1.24 (0.81–1.9)	0.312	1.15 (0.73–1.8)	0.550	1.85 (0.73–4.68)	0.190
≥500	1.12 (0.67–1.89)	0.661	0.99 (0.58–1.7)	0.980	1.2 (0.35–4.1)	0.780
Chemotherapy	0.51 (0.32–0.8)	0.003	0.42 (0.25–0.69)	0.001	0.16 (0.05–0.47)	0.001
R1 resection	1.68 (0.93–3.01)	0.084	1.59 (0.86–2.92)	0.140	0.87 (0.24–3.22)	0.840
Put a stent in	0.55 (0.27–1.13)	0.104	0.46 (0.22–0.97)	0.040	0.32 (0.04–2.3)	0.250
Biliary plasty	1.04 (0.69–1.57)	0.857	1.11 (0.71–1.72)	0.660	1.54 (0.57–4.13)	0.390
Postoperative complications	2.05 (1.43–2.92)	0.000	1.97 (1.36–2.84)	0.000	2.85 (1.21–6.74)	0.017

*HR* hazard ratio, *CI* confidence interval, *ASA* American Society of Anesthesiologists, *BMI* body mass index, *Tbil* total bilirubin, *AST* aspartate aminotransferase, *AJCC* American Joint Committee on Cancer, *LS* laparoscopic surgery, *COVID-19* coronavirus disease 2019

^a^In crude model, no covariates was being controlled in mixed-effect Cox model using the period of operation and the centers as a random effect

^b^In adjusted Cox model, covariates including age, gender, BMI, ASA score, preoperative Tbil, preoperative AST, preoperative Ca 19-9, tumor size, AJCC TNM stage, Bismuth-Corlett type, number of resected lymph nodes, operative time, transfusion during surgery, estimated blood loss, R0 resection, biliary plasty, stent placement, and chemotherapy being adjusted, followed by mixed-effect Cox model using the period of operation and the centers as a random effect

^c^In the propensity score-matched model, age, gender, BMI, ASA score, preoperative Tbil, preoperative AST, preoperative ALT, preoperative albumin, preoperative CA 19-9, tumor size, AJCC TNM stage, Bismuth-Corlett type, operative time, transfusion during surgery, estimated blood loss, number of resected lymph nodes, R0 resection, hepatectomy, conversion to laparotomy, vascular resection, digestive reconstruction, biliary plasty, stent placement, and chemotherapy were matched, followed by mixed-effect Cox model using the period of operation and the centers as a random effect

### Postoperative Outcomes According to Bismuth Type

In the present study, 226 patients with PHC had a low Bismuth type (Bismuth I/II) and 239 patients had a high Bismuth type (Bismuth III/IV), with significantly different surgical characteristics and OS. OS was much better in the low Bismuth type (median OS, NA; 95% CI 15–NA months) than in the high Bismuth type (median OS, 15; 95% CI 7–40 months, *p* < 0.0001) [electronic supplementary Table S2 and Fig. S2]. Furthermore, shorter LOS and less IBL were observed in patients who underwent LS compared with those who underwent OP among patients with Bismuth types I–II, with other characteristics being comparable between the groups. The OS was longer in the LS group than in the OP group, and the median OS was NA (IQR 20–NA) and 41 (95% CI 14–NA) months in both groups (*p* = 0.0469). Among patients with Bismuth types III–IV, those who underwent LS showed comparable or better short-term outcomes than the OP group, such as similar postoperative complications, shorter LOS (median, 14 vs. 17 days), and lower rates of severe complications (12.66% vs. 27.5%), demonstrating guaranteed safety of laparoscopic resection for high Bismuth types of PHC. However, a longer operation time, less vascular resection and biliary plasty, and higher postoperative hemorrhage were observed in the LS group. The OS was comparable between the LS and OP groups, with a median OS of 15 (IQR 8–NA) and 14 (95% CI 6–40) months in both groups (*p* = 0.4285) [electronic supplementary Fig. S3].

## Discussion

LS remains technically challenging and is usually performed in selected patients in a few high-volume centers. In the present study, we enrolled a large sample of patients with PHC who underwent LS, and compared the safety and long-term efficacy of LS with OP. Similar OS was observed between the two surgical groups in the well-matched cohort. However, a longer OS was observed in the LS group in the original cohort. The cause of such variation may be related to patient selection before surgery, which can be reflected by the baseline differences between the LS and OP groups, particularly significantly more females and better liver function in the LS group, and the demonstrated independent protective factors for OS in the original cohort. Assuming no selection bias, LS had similar postoperative short-term safety and long-term survival compared with OP, with evidence from multicentric practice. This also suggests that long-term survival can be achieved in some PHC patients, regardless of the surgical method.

Laparoscopic techniques were first used to determine the PHC staging and assess whether the tumor could be surgically resected.^34^ Recently, with the improvement of laparoscopic technology and instruments, combined with the accumulation of surgical experience and the improvement of surgical skills, the application of LS in PHC has been gradually developed and has shown promising short-term outcomes.^35,36^ Laparoscopy has the effect of magnification and blind area traversability, and thus has several advantages due to the clear visual field: the first, second, and third porta hepatis are more clearly exposed; convenient, highly selective separation of blood vessels entering and exiting the liver; and identification of the variant hepatic artery, portal vein, and bile duct. Theoretically, LS can provide satisfactory safety during complex surgical procedures. However, the complex location of PHC, adjacent to the hepatic artery, portal vein, and hepatic parenchyma, and the complex surgical resections for PHC, including liver resection, bilio-enteric reconstruction, radical lymph node dissection around the perihilar, retropancreatic, and para-aortic areas, as well as the high incidence of postoperative complications, have hindered the development of LS in PHC, which has been far behind other abdominal surgeries. In the present study, LS showed a similar R0 resection rate, lymph node retrieval, and operative time as OP. The LOS was shorter and the incidence of severe postoperative complications (CD >III) was lower in the LS group. All the evidence presented the acceptability of LS in PHC applications to obtain optimized short-term safety.

Laparoscopy in PHC evolved rapidly during the study period (from 2013 to 2018). Most notably, the number of PHC patients with stage III/IV LS increased annually during the study period from 25 to 60%. The attention of surgical improvement towards PHC has focused on the technical methods of controlling microscopic spread of disease to achieve long-term survival and optimize short-term surgical results.^37,38^ Most previous studies have guaranteed short-term outcomes. However, the paucity of reports in the literature focusing on long-term evidence suggests that a step towards the systematization of the laparoscopic approach has not yet been taken due to a conceptual barrier. A recent analysis of patients with PHC treated in one of the leading European centers for LS showed no oncologic inferiority of laparoscopic resection, which posed a major concern to surgeons preparing for the final step toward minimally invasive PHC surgery.^39^ In the present study, the median OS in the LS group was higher than that in the OP group, suggesting better survival following LS for some well-selected patients.

The complexity of surgical procedures for PHC and survival is mainly dependent on the Bismuth–Corlett type. Laparoscopy has been used for all Bismuth types, although it is predominantly used in patients with low-stage PHCs.^40–42^ For PHC patients with high Bismuth type (III/IV), surgery involves caudate lobectomy, complex biliojejunal anastomosis, multiple biliary tract reconstruction, and vascular reconstruction, which dramatically increases the complexity of the operation.^43^ Laparoscopic caudate lobe resection is a feasible and safe procedure.^44,45^ The three-dimensional visualization technique can be used to accurately evaluate the scope of tumor status as well as the invasion status of peripheral blood vessels and bile ducts. Therefore, it is possible to develop a detailed surgical plan using multidisciplinary instructions for complex surgery.^46^ In addition, through external liver suspension during the operation, the hilum can be better exposed, and the operation field can significantly expand.^47^ To date, several attempts have been made to perform laparoscopic resection of the Bismuth III/IV type, and some promising short-term clinical outcomes have been achieved. In the present study, LS showed comparable or better short-term outcomes in PHC with Bismuth III/IV type compared with OP, such as similar postoperative complications, shorter LOS, and lower severity of complications, indicating the guaranteed safety of laparoscopic resection for PHC with high Bismuth classification. However, the longer operation time in LS than in OP indicates that the LS for PHC is still in its learning stage.

Postoperative adjuvant radiotherapy and chemotherapy have become the major treatment options for malignant tumors. However, the role of adjuvant therapy and the exact postoperative regimens for PHC remain controversial, with no prospective randomized controlled studies on this issue at present. In the 2019 American Society of Clinical Oncology (ASCO) clinical practice guidelines, oral capecitabine was recommended as adjuvant chemotherapy following surgery for patients with resected biliary tract cancer, based on the results of the BILCAP randomized controlled trial; however, subgroup analysis of patients with PHC failed to yield positive results.^48^ Some retrospective reports have suggested that adjuvant treatment could improve the OS of patients with PHC with lymph node metastases^49,50^ or with positive resection margins.^51^ Based on the ASCO clinical practice guidelines, adjuvant chemoradiotherapy is recommended for patients with PHC who undergo R1 resection.^52^ The present study suggests that adjuvant chemotherapy is a significant protective prognostic factor for long-term survival in patients with PHC. Subgroup analyses of Bismuth type III–IV patients with R0 resection revealed that adjuvant chemotherapy produced significant survival benefits when compared with surgery alone (electronic supplementary Fig. S4); a similar result was reported by Im et al.^53^ However, a relatively low percentage of patients received adjuvant chemotherapy and there was a large difference in chemotherapy percentages between the LS and OP groups. A previous study demonstrated that LS is associated with greater rates of compliance with guidelines for adjuvant chemotherapy, as well as a slightly shorter time before initiation of chemotherapy.^54^ Faster postoperative recovery and decreased postoperative complications could increase the likelihood of receiving adjuvant chemotherapy after LS compared with OP. With the cumulative evidence supporting the necessity for adjuvant therapy, improving compliance to guidelines for adjuvant chemotherapy and reaching a consensus regarding the detailed implementation plan for adjuvant treatment needs to be further validated through strict postoperative patient management in future studies. Other risk factors such as elevated CA19-9 level reflecting higher tumor burden,^55,56^ elevated AST level reflecting poor liver status,^57^ and older age reflecting poor physical function, were all associated with poor survival outcomes. Surgical resection with negative histologic margins (R0 resection) was considered the only option for long-term survival in patients with PHC.^58,59^ The R0 rates in the LS and OP groups were 91.3% and 91.83%, respectively. The high negative resection rate in this analysis guarantees postoperative benefit for patients with PHC in both the LS and OP groups.

The results for the overall experience were positive; however, the methodological and technical limitations of the present study may be due to its retrospective nature and small sample size. The LS and OP cases were obtained from 10 institutions, and potential selection bias could not be avoided. In addition, the relatively short follow-up period, especially in the LS group, limited sufficient analyses of long-term survival between the LS and OP groups. Additionally, oncological outcomes, such as recurrence-free survival or disease-free survival, were not documented; therefore, we could not thoroughly compare the disease progression process between the two surgical groups. Finally, with the increasing popularity of robotic platforms for complex hepatic resections and reconstructions worldwide,^60^ we did not include the robotic surgeries because of the relatively small number of cases during the research period. In future studies, we will include more patients who underwent different types of PHC surgeries to further investigate individualized surgical treatment strategies.

## Conclusions

LS could be technically feasible and achieve equivalent long-term survival as OP in patients with resectable PHC, regardless of the Bismuth–Corlett type. Additionally, LS can shorten the length of hospital stay and reduce the occurrence of severe postoperative complications. However, considering the steep learning curve and high risks involved, this procedure should be performed by experienced surgeons after adequate training in high-volume laparoscopic liver centers.

## Supplementary Information

Below is the link to the electronic supplementary material.

**Supplementary Fig. S1** Random effects. (**a**) random effects of centers. (**b**) Random effects between surgery year. 95% CIs shown

**Supplementary Fig. S2** Kaplan–Meier curves for over survival of PHC patients with different Bismuth type. (**a**) Before propensity score matching; (**b**) propensity score matching. *LS* laparoscopic surgery, *OP* open operation, *HR* hazard ratio, *CI* confidence interval

**Supplementary Fig. S3** Kaplan–Meier curves for over survival of PHC patients undergoing LS or OP with different Bismuth type. (**a**) Bismuth I/II type before propensity score matching. (**b**) Bismuth I/II type after propensity score matching. (**c**) Bismuth III/IV type before propensity score matching. (**d**) Bismuth III/IV type after propensity score matching. *LS* laparoscopic surgery, *OP* open operation, *HR* hazard ratio, *CI* confidence interval

**Supplementary Fig. S4** Kaplan–Meier curves for over survival of PHC patients with Bismuth type III-IV patients with R0 resection. (**a**) Before propensity score matching. (**b**) propensity score matching. *LS* laparoscopic surgery, *OP* open operation, *HR* hazard ratio, *CI* confidence interval

**Supplementary Table S1** The year-specific survival rate of LS and OP for PHC subgroups in raw data.

**Supplementary Table S2** The interoperative and postoperative characteristics according to Bismuth type for PHC patients.

**Supplementary Table S3** The interoperative and postoperative characteristics according to surgical method in Bismuth type I/II and Bismuth type III/IV, respectively.
