# Supplementary figures and images for: The Long-Term Outcome of Laparoscopic Resection for Perihilar Cholangiocarcinoma Compared with the Open Approach: A Real-World Multicentric Analysis
Source: Ann Surg Oncol. 2022 Oct 22;30(3):1366–78. doi: 10.1245/s10434-022-12647-1 (PMC9589740; doi:10.1245/s10434-022-12647-1)

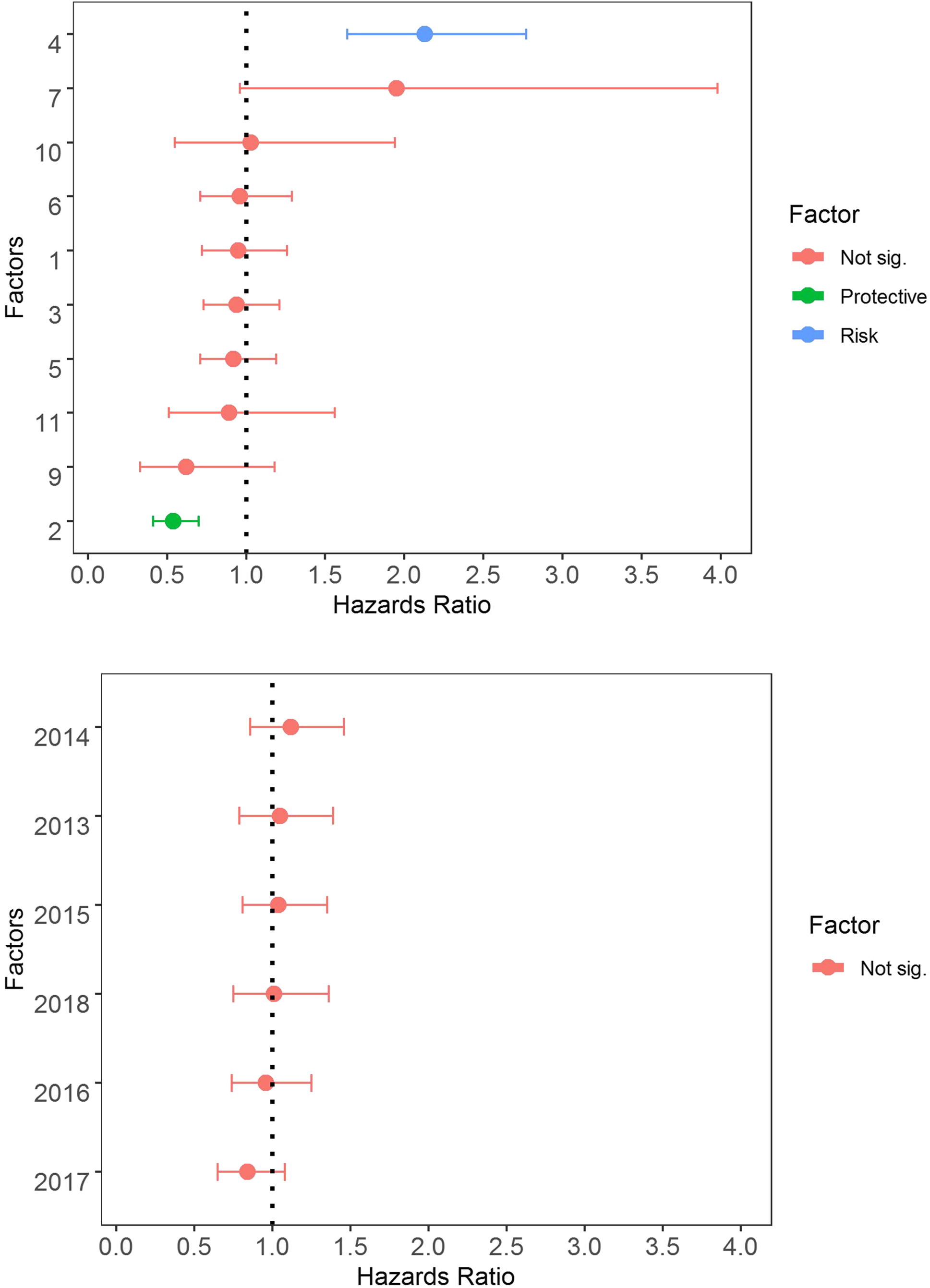

Supplement: Supplementary file 1 — Supplementary Fig. S1 Random effects. (a) random effects of centers. (b) Random effects between surgery year. 95% CIs shown [file 10434_2022_12647_MOESM1_ESM.tif]

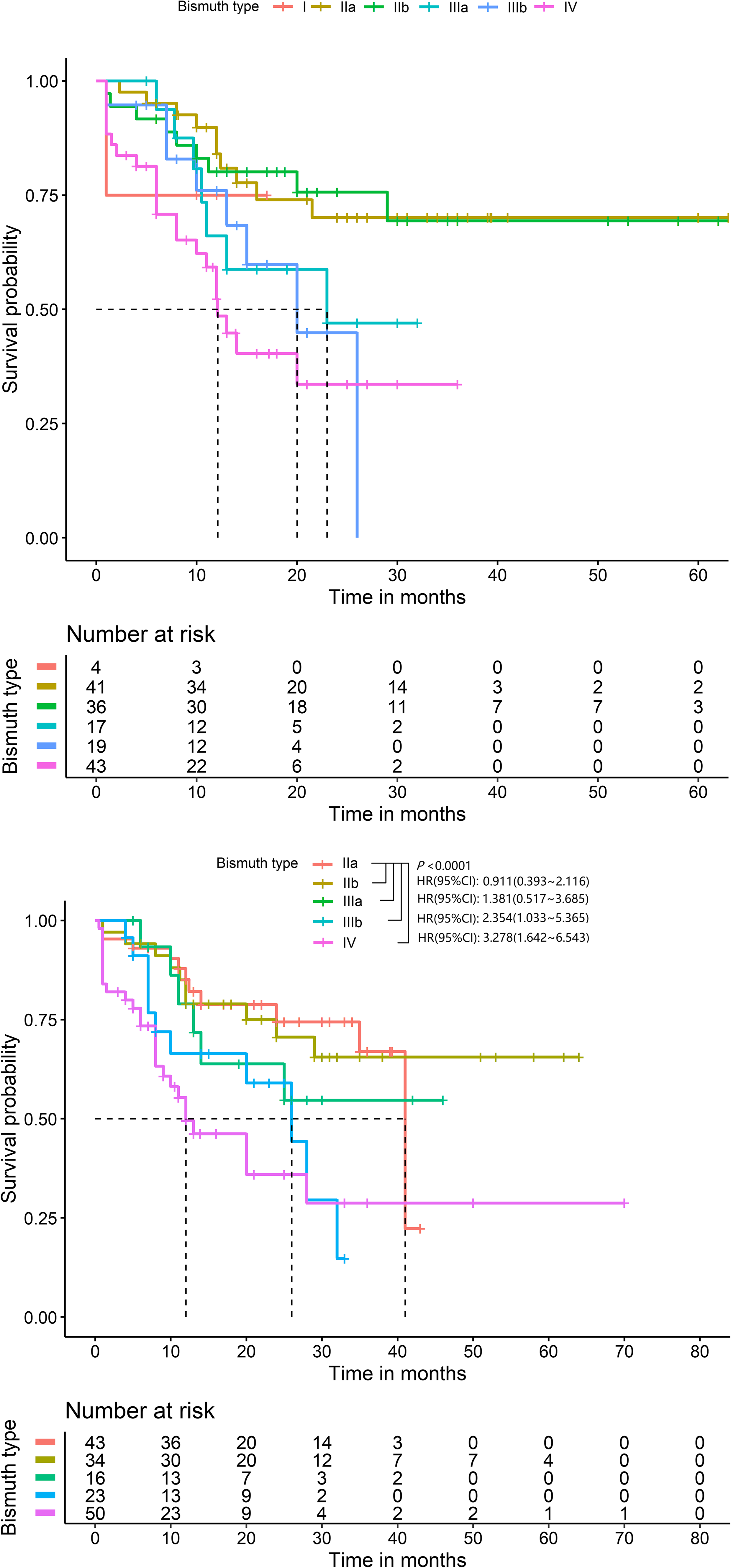

Supplement: Supplementary file 2 — Supplementary Fig. S2 Kaplan–Meier curves for over survival of PHC patients with different Bismuth type. (a) Before propensity score matching; (b) propensity score matching. LS laparoscopic surgery, OP open operation, HR hazard ratio, CI confidence interval [file 10434_2022_12647_MOESM2_ESM.tif]

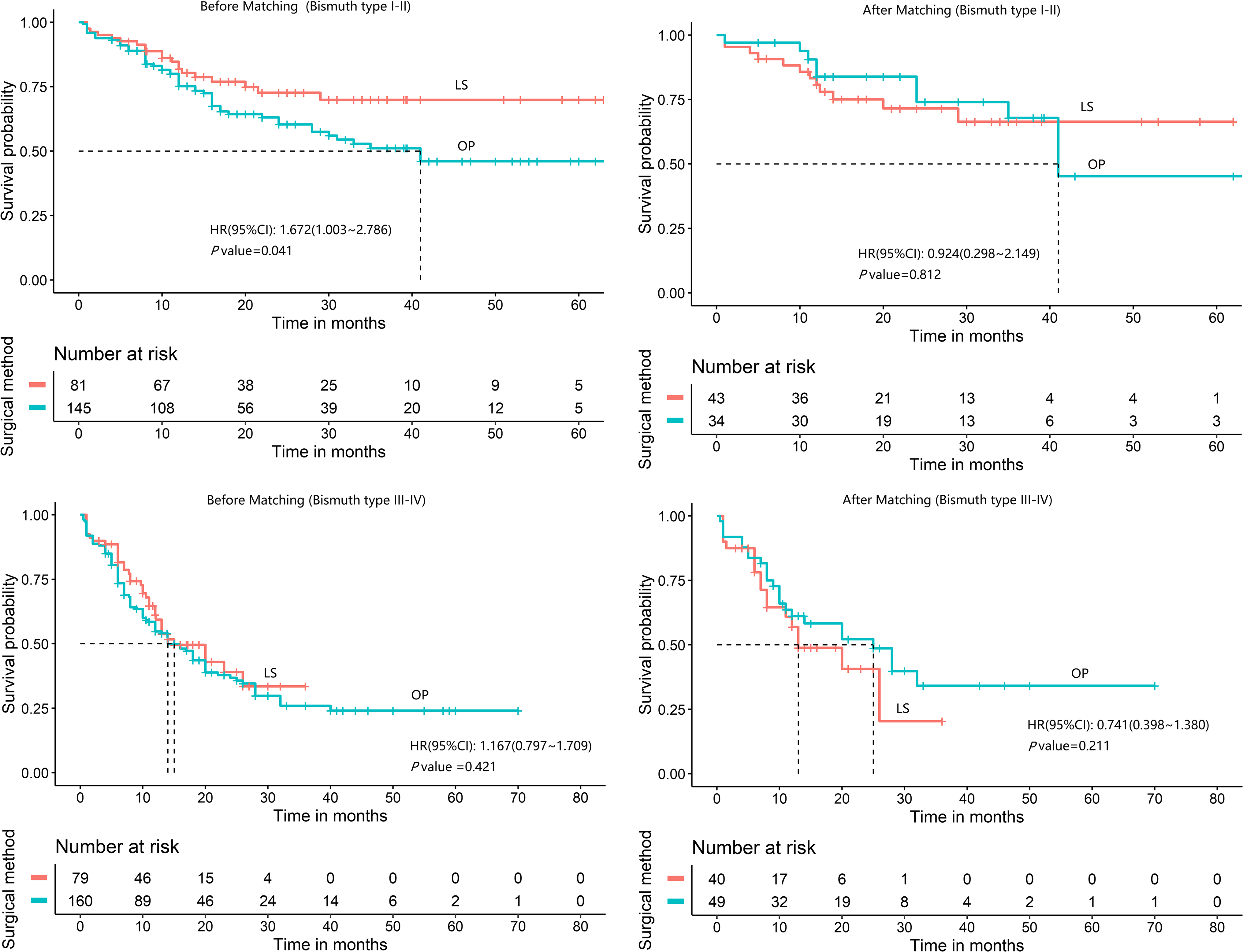

Supplement: Supplementary file 3 — Supplementary Fig. S3 Kaplan–Meier curves for over survival of PHC patients undergoing LS or OP with different Bismuth type. (a) Bismuth I/II type before propensity score matching. (b) Bismuth I/II type after propensity score matching. (c) Bismuth III/IV type before propensity score matching. (d) Bismuth III/IV type after propensity score matching. LS laparoscopic surgery, OP open operation, HR hazard ratio, CI confidence interval [file 10434_2022_12647_MOESM3_ESM.tif]

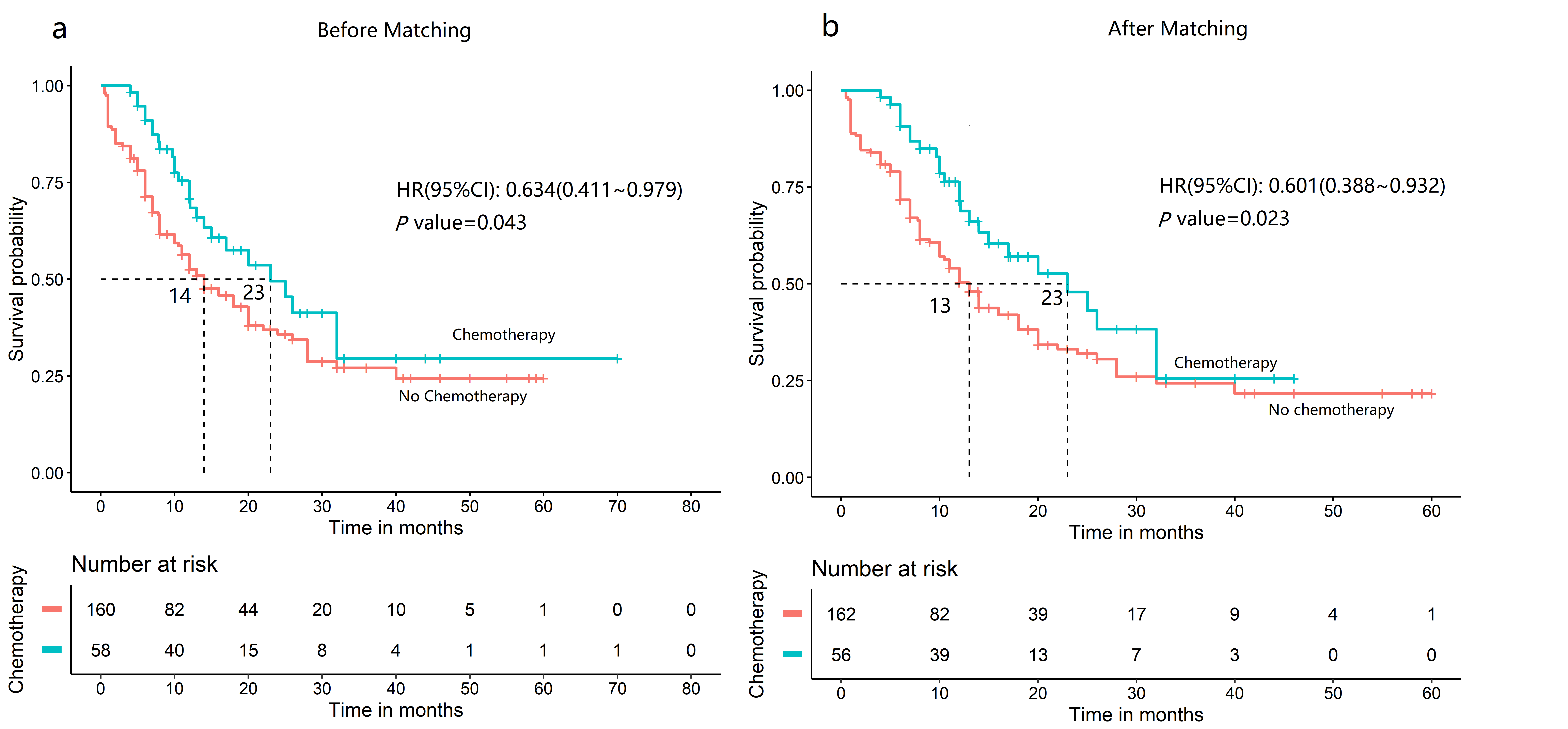

Supplement: Supplementary file 4 — Supplementary Fig. S4 Kaplan–Meier curves for over survival of PHC patients with Bismuth type III-IV patients with R0 resection. (a) Before propensity score matching. (b) propensity score matching. LS laparoscopic surgery, OP open operation, HR hazard ratio, CI confidence interval [file 10434_2022_12647_MOESM4_ESM.jpg]
