# Supplementary material for: The Long-Term Outcome of Laparoscopic Resection for Perihilar Cholangiocarcinoma Compared with the Open Approach: A Real-World Multicentric Analysis
Source: Ann Surg Oncol. 2022 Oct 22;30(3):1366–78. doi: 10.1245/s10434-022-12647-1 (PMC9589740; doi:10.1245/s10434-022-12647-1)
Supplement: Supplementary file 5 — Supplementary Table S1 The year-specific survival rate of LS and OP for PHC subgroups in raw data. [file 10434_2022_12647_MOESM5_ESM.docx]

**Supplementary table 1.** **The year-specific survival rate of LS and OP for PHC subgroups in raw data.**

|  |  | **Before Matching** | | | | |  |  | **After Matching** | | | | |
| --- | --- | --- | --- | --- | --- | --- | --- | --- | --- | --- | --- | --- | --- |
| **Characteristic** |  | **1- year Survival Rate** | |  | **3- year Survival Rate** | |  |  | **3- year Survival Rate** | |  | **3- year Survival Rate** | |
|  | **N** | **LS** | **OP** |  | **LS** | **OP** |  | **N** | **LS** | **OP** |  | **LS** | **OP** |
| **Raw Data** |  |  |  |  |  |  |  |  |  |  |  |  |  |
| Gender |  |  |  |  |  |  |  |  |  |  |  |  |  |
| Male | 274 | 0.66(0.53,0.75) | 0.62(0.54,0.69) |  | 0.43(0.28,0.57) | 0.34(0.25,0.42) |  | 82 | 0.69(0.52,0.81) | 0.64(0.46,0.77) |  | 0.47(0.26,0.66) | 0.29(0.10,0.53) |
| Female | 193 | 0.79(0.68,0.87) | 0.69(0.59,0.76) |  | 0.66(0.52,0.77) | 0.44(0.33,0.54) |  | 84 | 0.72(0.54,0.84) | 0.76(0.60,0.87) |  | 0.58(0.36, 0.74) | 0.60(0.42,0.74) |
| Age, year |  |  |  |  |  |  |  |  |  |  |  |  |  |
| <60 | 155 | 0.76(0.61,0.87) | 0.76(0.66,0.83) |  | 0.54(0.32,0.71) | 0.50(0.37,0.61) |  | 57 | 0.72(0.50,0.86) | 0.86(0.66,0.94) |  |  | 0.70(0.42,0.87) |
| ≥60 | 312 | 0.70(0.60,0.78) | 0.59(0.51,0.65) |  | 0.54(0.43,0.65) | 0.32(0.24,0.40) |  | 109 | 0.70(0.54,0.81) | 0.63(0.48,0.74) |  | 0.54(0.37,0.69) | 0.38(0.22,0.54) |
| BMI, Kg/cm^2^ |  |  |  |  |  |  |  |  |  |  |  |  |  |
| <18.5 | 20 | 0.57(0.22,0.81) | 0.63(0.22,0.87) |  | 0.57(0.22,0.81) | 0.32(0.05,0.65) |  | 7 | 0.63(0.14,0.89) |  |  |  |  |
| 18.5~24 | 322 | 0.76(0.66,0.84) | 0.65(0.58,0.71) |  | 0.57(0.43,0.69) | 0.40(0.32,0.47) |  | 110 | 0.75(0.59,0.85) | 0.68(0.53,0.79) |  | 0.52(0.31, 0.69) | 0.43(0.27,0.59) |
| >24 | 125 | 0.68(0.53,0.79) | 0.63(0.51,0.73) |  | 0.49(0.32,0.64) | 0.32(0.18,0.46) |  | 49 | 0.63(0.40,0.80) | 0.75(0.53,0.88) |  | 0.49(0.25, 0.70) | 0.60(0.33,0.79) |
| Bismuth-Corlett stage |  |  |  |  |  |  |  |  |  |  |  |  |  |
| I - II | 226 | 0.82(0.71,0.89) | 0.75(0.67,0.82) |  | 0.70(0.56,0.80) | 0.51(0.40,0.61) |  | 77 | 0.81(0.65,0.90) | 0.84(0.65,0.93) |  | 0.66(0.47, 0.80) | 0.68(0.44,0.83) |
| III - IV | 239 | 0.61(0.48,0.72) | 0.55(0.46,0.62) |  | 0.33(0.18,0.49) | 0.26(0.18,0.35) |  | 89 | 0.57(0.38,0.72) | 0.61(0.46,0.74) |  | 0.20(0.02, 0.53) | 0.34(0.17,0.52) |
| Resection |  |  |  |  |  |  |  |  |  |  |  |  |  |
| R0 | 428 | 0.73(0.64,0.79) | 0.66(0.60,0.71) |  | 0.54(0.44,0.64) | 0.40(0.32,0.47) |  |  | 0.73(0.61,0.82) | 0.70(0.57, 0.79) |  | 0.55(0.39, 0.68) | 0.49(0.34,0.63) |
| R1 | 39 | 0.67(0.33,0.86) | 0.51(0.29,0.70) |  | 0.57(0.25,0.80) | 0.17(0.04,0.40) |  |  | 0.40(0.05,0.75) | 0.78(0.36, 0.94) |  |  | 0.41(0.07,0.75) |
| Year of operation |  |  |  |  |  |  |  |  |  |  |  |  |  |
| 2013–2014 | 94 | 0.59(0.37,0.75) | 0.54(0.41,0.65) |  | 0.42(0.22,0.60) | 0.33(0.23,0.44) |  |  | 0.55(0.23,0.78) | 0.53(0.28, 0.72) |  | 0.36(0.11, 0.63) | 0.42(0.20,0.62) |
| 2015–2016 | 166 | 0.70(0.56,0.80) | 0.65(0.55,0.73) |  | 0.56(0.41,0.68) | 0.37(0.28,0.46) |  |  | 0.71(0.51,0.86) | 0.73(0.56, 0.84) |  | 0.56(0.35, 0.72) | 0.45(0.26,0.62) |
| 2017–2018 | 207 | 0.78(0.65,0.86) | 0.72(0.63,0.80) |  |  |  |  |  | 0.74(0.55,0.86) | 0.80(0.55, 0.92) |  |  |  |
| AJCC TNM stage |  |  |  |  |  |  |  |  |  |  |  |  |  |
| I(T1N0M0) | 78 | 0.75(0.46, 0.90) | 0.79(0.65, 0.87) |  |  | 0.56(0.38,0.70) |  |  | 0.74(0.39,0.91) | 0.86(0.53, 0.96) |  |  | 0.57(0.19,0.83) |
| II(T2a/2bN0M0) | 195 | 0.70(0.58,0.80) | 0.70(0.61,0.78) |  | 0.58(0.44,0.70) | 0.34(0.22,0.45) |  |  | 0.74(0.55,0.85) | 0.72(0.55, 0.84) |  | 0.62(0.42, 0.77) | 0.39(0.19,0.59) |
| III(T3/4N0M0) | 75 | 0.84(0.68,0.92) | 0.56(0.38,0.70) |  | 0.55(0.34,0.72) | 0.42(0.26,0.58) |  |  | 0.68(0.35,0.87) | 0.73(0.37, 0.90) |  | 0.41(0.11, 0.70) |  |
| IV(N2M0/N,M1) | 108 | 0.56(0.34,0.74) | 0.47(0.36,0.58) |  | 0.51(0.28,0.69) | 0.20(0.10,0.31) |  |  | 0.61(0.33,0.81) | 0.52(0.26, 0.72) |  |  | 0.44(0.20,0.66) |
| Tumor size |  |  |  |  |  |  |  |  |  |  |  |  |  |
| <3 | 314 | 0.71(0.60,0.79) | 0.68(0.61,0.74) |  | 0.50(0.38,0.62) | 0.41(0.32,0.50) |  |  | 0.67(0.52,0.78) | 0.68(0.53, 0.79) |  | 0.49(0.31, 0.65) | 0.46(0.29, 0.61) |
| ≥3 | 153 | 0.75(0.60,0.85) | 0.56(0.45,0.66) |  | 0.63(0.46,0.76) | 0.31(0.20,0.43) |  |  | 0.78(0.54,0.90) | 0.75(0.54, 0.87) |  | 0.60(0.34,0.78) | 0.51(0.24.0.73) |
