# Supplementary material for: The Long-Term Outcome of Laparoscopic Resection for Perihilar Cholangiocarcinoma Compared with the Open Approach: A Real-World Multicentric Analysis
Source: Ann Surg Oncol. 2022 Oct 22;30(3):1366–78. doi: 10.1245/s10434-022-12647-1 (PMC9589740; doi:10.1245/s10434-022-12647-1)
Supplement: Supplementary file 6 — Supplementary Table S2 The interoperative and postoperative characteristics according to Bismuth type for PHC patients. [file 10434_2022_12647_MOESM6_ESM.docx]

Supplementary table 2. The interoperative and postoperative characteristics according to Bismuth type for PHC patients.

| Variable | Bismuth I/II (N=226) | Bismuth III/IV (N=239) | P value |
| --- | --- | --- | --- |
| Operative time, min, Median (IQR) | 309.0(251.5~390.0) | 360.0(320.0~420.0) | <0.0001 |
| Resection time, min, Median (IQR) | 230.0(195.0~315.0) | 230.0(220.0~270.0) | 0.1967 |
| Anastomosis & reconstruction time, min, Median (IQR) | 45.0(35.0~120.0) | 130.0(45.0~150.0) | <0.0001 |
| IBL, ml, Median (IQR) | 200.0(100.0~300.0) | 400.0(200.0~600.0) | <0.0001 |
| Transfusion during surgery, No.(%) | 57(25.22) | 90(37.7) | 0.004 |
| Transfusion colume, ml, Median (IQR) | 0.0(0.0~400.0) | 0.0(0.0~600.0) | 0.003 |
| Maximun tumor size, mm, mean(SD) | 2.62(1.2) | 3.16(1.3) | <0.0001 |
| No. of harvested lymph nodes, Median (IQR) | 6.0(4.0~8.0) | 8.0(5.0~10.0) | 0.003 |
| PDTK, day, median(IQR) | 8.0(5.0~12.0) | 8.0(5.0~14.0) | 0.058 |
| Postoperative hospital stay, day, Median(IQR) | 13.0(11.0~18.0) | 16.0(13.0~23.0) | <0.0001 |
| Vascular resection, No. (%) |  |  |  |
| None | 201(88.9) | 177(74.1) | <0.0001 |
| Hepatic artery | 15(6.6) | 12(5.0) |  |
| Portal vein | 3(1.3) | 9(3.8) |  |
| Hepatic artery & Portal vein | 7(3.1) | 41(17.2) |  |
| Biliary plasty, No. (%) | 88(38.9) | 141(59.0) | <0.0001 |
| Put a stent, No.(%) | 23(10.2) | 11(4.6) | 0.021 |
| Major complications, No. (%) |  |  |  |
| Biliary fistula | 8(3.5) | 28(11.7) | 0.001 |
| Hemorrhage | 8(3.5) | 18(7.5) | 0.061 |
| Abdominal abscess | 22(9.7) | 32(13.4) | 0.219 |
| Gastrointestinal fistula | 3(1.3) | 1(0.4) | 0.289^*^ |
| Incision infection | 5(2.2) | 9(3.8) | 0.327 |
| Pneumonia | 13(5.8) | 27(11.3) | 0.033 |
| Renal failure | 4(1.8) | 9(3.8) | 0.192 |
| Heart failure | 5(2.2) | 11(4.6) | 0.158 |
| Liver failure | 1(0.4) | 11(4.6) | 0.005^*^ |
| ARDS | 4(1.8) | 8(3.4) | 0.284 |
| Reoperation, No. (%) | 7(3.1) | 5(2.1) | 0.494 |
| R0, No. (%) | 16(7.1) | 21(8.8) | 0.497 |
| TNM Stage, No. (%) |  |  | <0.0001 |
| I(T1N0M0) | 62(28.3) | 16(6.8) |  |
| II(T2a/2bN0M0) | 95(43.4) | 100(42.6) |  |
| IIIA(T3N0M0) | 16(7.3) | 30(12.8) |  |
| IIIB(T4N0M0) | 11(5.0) | 18(7.7) |  |
| IVA(T, N2M0) | 23(10.5) | 51(21.7) |  |
| IVB(T,N,M1) | 12(5.5) | 20(8.5) |  |
| Death (30d), No. (%) | 9(4.0) | 20(8.4) | 0.051 |
| Death (90d), No. (%) | 14(6.2) | 27(11.3) | 0.052 |
| CD stage ≥ III, No. (%) | 41(18.1) | 54(22.6) | 0.234 |
| OS, month, median (95%CI) | NA(15~NA) | 15(7~40) | <.0001 |

PHC, perihilar cholangiocarcinoma; IBL, intraoperative blood loss; PDTK, postoperative drainage tube keep time; ARDS, Acute Respiratory Distress Syndrome; CD, Clavien-Dindo; OS, overall survival.

*Fisher exact test.
