# Supplementary material for: The Long-Term Outcome of Laparoscopic Resection for Perihilar Cholangiocarcinoma Compared with the Open Approach: A Real-World Multicentric Analysis
Source: Ann Surg Oncol. 2022 Oct 22;30(3):1366–78. doi: 10.1245/s10434-022-12647-1 (PMC9589740; doi:10.1245/s10434-022-12647-1)
Supplement: Supplementary file 7 — Supplementary Table S3 The interoperative and postoperative characteristics according to surgical method in Bismuth type I/II and Bismuth type III/IV, respectively. [file 10434_2022_12647_MOESM7_ESM.docx]

Supplementary table 3. The interoperative and postoperative characteristics according to LS and OP among PHC patients with different Bismuth type.

| **Variable** | Bismuth I/II (N=226) | | |  | Bismuth III/IV (N=239) | | |
| --- | --- | --- | --- | --- | --- | --- | --- |
|  | LS(N=81) | OP (N=145) | P value |  | LS (N=79) | OP (N=160) | P value |
| Operative time, min, Median (IQR) | 330.0(260.0~420.0) | 300.0(240.0~370.0) | 0.179 |  | 360.0(303.0~430.0) | 360.0(320.0~400.0) | 0.052 |
| Resection time, min, Median (IQR) | 255.0(205.0~350.0) | 220.0(180.0~260.0) | 0.203 |  | 265.0(220.0~325.0) | 220.0(200.0~240.0) | <0.0001 |
| Anastomosis time, min, Median (IQR) | 40.0(35.0~45.0) | 100.0(35.0~120.0) | <0.0001 |  | 40.0(34.0~130.0) | 140.0(120.0~160.0) | <0.0001 |
| Transfusion during surgery, No.(%) | 13(16.1) | 44(30.3) | 0.018 |  | 31(39.2) | 59(36.9) | 0.723 |
| IBL, ml, Median (IQR) | 200.0(100.0~300.0) | 200.0(100.0~400.0) | 0.271 |  | 400.0(200.0~700.0) | 400.0(200.0~500.0) | 0.404 |
| Transfusion colume, ml, Median (IQR) | 0.0(0.0~0.0) | 0.0(0.0~400.0) | 0.047 |  | 0.0(0.0~600.0) | 0.0(0.0~690.0) | 0.305 |
| Maximun tumor size, mm, mean(SD) | 2.8(1.3) | 2.6(1.2) | 0.206 |  | 3.1(1.2) | 3.2(1.4) | 0.643 |
| No. of harvested lymph nodes, Median (IQR) | 6.0(3.0~8.0) | 7.0(5.0~8.0) | 0.162 |  | 6.0(5.0~9.0) | 8.0(6.0~10.0) | 0.083 |
| PDTK, day, median(IQR) | 7.0(4.0~10.0) | 9.0(5.0~13.0) | 0.028 |  | 9.0(5.0~12.0) | 8.0(5.0~15.0) | 0.253 |
| Postoperative hospital stay, day, Median(IQR) | 12.0(9.0~14.0) | 14.0(12.0~19.0) | 0.011 |  | 14.0(12.0~20.0) | 17.0(14.0~24.5) | 0.018 |
| Vascular resection, No. (%) |  |  |  |  |  |  |  |
| None | 76(93.8) | 125(86.2) | 0.187^*^ |  | 72(91.1) | 105(65.6) | <0.0001^*^ |
| Hepatic artery | 4(4.9) | 11(7.6) |  |  | 5(6.3) | 7(4.4) |  |
| Portal vein | 1(1.2) | 2(1.4) |  |  | 2(2.5) | 7(4.4) |  |
| Hepatic artery & Portal vein | 0(0.0) | 7(4.8) |  |  | 0(0.0) | 41(25.6) |  |
| Biliary plasty, No. (%) | 18(22.2) | 70(48.3) | <0.0001 |  | 38(48.1) | 103(64.4) | 0.016 |
| Put a stent, No. (%) | 8(9.9) | 15(10.3) | 0.911 |  | 2(2.5) | 9(5.6) | 0.283 |
| Choledochojejunostomy, No.(%) |  |  |  |  |  |  |  |
| No suture | 7(8.6) | 4(2.8) | <0.0001^*^ |  | 7(8.9) | 4(2.5) | <0.0001^*^ |
| Continuous suture | 60(74.1) | 34(23.5) |  |  | 50(63.3) | 38(23.8) |  |
| Interrupted suture | 13(16.1) | 78(53.8) |  |  | 19(24.1) | 93(58.1) |  |
| Combined suture | 1(1.2) | 29(20.0) |  |  | 3(3.8) | 25(15.6) |  |
| Major complications, No. (%) |  |  |  |  |  |  |  |
| Biliary fistula | 4(4.9) | 4(2.8) | 0.395 |  | 9(11.4) | 19(11.9) | 0.913 |
| Hemorrhage | 2(2.5) | 6(4.1) | 0.515 |  | 11(13.9) | 7(4.4) | 0.009 |
| Abdominal abscess | 6(7.4) | 16(11.0) | 0.378 |  | 8(10.1) | 24(15.0) | 0.298 |
| Gastrointestinal fistula | 1(1.2) | 2(1.4) | 0.927^*^ |  | 0(0.0) | 1(0.6) | 0.481^*^ |
| Incision infection | 1(1.2) | 4(2.8) | 0.455^*^ |  | 1(1.3) | 8(5.0) | 0.154^*^ |
| Pneumonia | 6(7.4) | 7(4.8) | 0.424 |  | 7(8.9) | 20(12.5) | 0.403 |
| Renal failure | 1(1.2) | 3(2.1) | 0.648^*^ |  | 2(2.5) | 7(4.4) | 0.481^*^ |
| Heart failure | 1(1.2) | 4(2.8) | 0.455^*^ |  | 1(1.3) | 10(6.3) | 0.084^*^ |
| Liver failure | 0(0.0) | 1(0.7) | 0.454^*^ |  | 3(3.8) | 8(5.0) | 0.676 |
| ARDS | 1(1.2) | 3(2.1) | 0.648^*^ |  | 2(2.5) | 6(3.8) | 0.622 |
| Reoperation, No. (%) | 2(2.5) | 5(3.5) | 0.684 |  | 3(3.8) | 2(1.3) | 0.196 |
| R0, No. (%) | 6(7.4) | 10(6.9) | 0.886 |  | 7(8.9) | 14(8.8) | 0.977 |
| TNM Stage, No. (%) |  |  |  |  |  |  |  |
| I(T1N0M0) | 14(18.2) | 48(33.8) | 0.040 |  | 5(6.3) | 11(7.1) | 0.007 |
| II(T2a/2bN0M0) | 38(49.4) | 57(40.1) |  |  | 35(44.3) | 65(41.7) |  |
| IIIA(T3N0M0) | 7(9.1) | 9(6.3) |  |  | 18(22.8) | 12(7.7) |  |
| IIIB(T4N0M0) | 7(9.1) | 4(2.8) |  |  | 7(8.9) | 11(7.1) |  |
| IVA(T, N2M0) | 9(11.7) | 14(9.9) |  |  | 10(12.7) | 41(26.3) |  |
| IVB(T,N,M1) | 2(2.6) | 10(7.0) |  |  | 4(5.1) | 16(10.3) |  |
| Death (30d), No. (%) | 2(2.5) | 7(4.8) | 0.385 |  | 6(7.6) | 14(8.8) | 0.762 |
| Death (90d), No. (%) | 4(4.9) | 10(6.9) | 0.558 |  | 8(10.1) | 19(11.9) | 0.688 |
| CD stage ≥ III, No. (%) | 10(12.4) | 31(21.4) | 0.091 |  | 10(12.7) | 44(27.5) | 0.010 |
| Year of operation, No. (%) |  |  |  |  |  |  |  |
| 2013–2014 | 15(18.5) | 30(20.7) | 0.926 |  | 10(12.7) | 39(24.4) | 0.007 |
| 2015–2016 | 30(37.0) | 52(35.9) |  |  | 23(29.1) | 61(38.1) |  |
| 2017–2018 | 36(44.4) | 63(43.5) |  |  | 46(58.2) | 60(37.5) |  |
| OS, month, median (95%CI) | NA(20~NA) | 41(14~NA) | 0.0469 |  | 15(8~NA) | 14(6~40) | 0.429 |

PHC, perihilar cholangiocarcinoma; IQR, interquartile range; SD, standard deviation; IBL, intraoperative blood loss; PDTK, postoperative drainage tube keep time; ARDS, Acute Respiratory Distress Syndrome; CD, Clavien-Dindo; OS, overall survival.
